# Complete Genome Sequence of a Strain of *Bifidobacterium pseudolongum* Isolated from Mouse Feces and Associated with Improved Organ Transplant Outcome

**DOI:** 10.1128/genomeA.01089-17

**Published:** 2017-10-05

**Authors:** Emmanuel F. Mongodin, Lauren L. Hittle, Suvarna Nadendla, C. Colin Brinkman, Yanbao Xiong, Jonathan S. Bromberg

**Affiliations:** a Institute for Genome Sciences, University of Maryland School of Medicine, Baltimore, Maryland, USA; b Department of Surgery, University of Maryland School of Medicine, Baltimore, Maryland, USA

## Abstract

Here, we report the complete genome sequence of *Bifidobacterium pseudolongum* strain UMB-MBP-01, isolated from the feces of C57BL/6J mice. This strain was identified in microbiome profiling studies and associated with improved transplant outcome in a murine model of cardiac heterotypic transplantation.

## GENOME ANNOUNCEMENT

Members of the *Bifidobacterium* genus are high-G+C Gram-positive bacteria that were first isolated and described over a century ago from the feces of breastfed infants (1). Since then, *Bifidobacterium* species have been isolated from the digestive tracts of various mammalian species, including human and mouse (2–4), insects (5), and birds (6), as well as from sewage and food, these two ecological niches being linked to contamination originating from the human/animal intestinal environments (6, 7). Because of their positive health benefits to the human host, *Bifidobacterium* bacteria have been the intense focus of industrial and scientific interests, mostly for their potential use as probiotics. However, the specific molecular cascades involved in the *Bifidobacterium*-host cross talks promoting these beneficial health effects remain largely unknown (8, 9).

The use of *in vivo* murine models to characterize the impact of specific members of the intestinal microbiota on the host physiology is a promising avenue for dissecting the key pathways involved in these interactions (3, 10). Among bifidobacteria, *Bifidobacterium pseudolongum* has been described as one of the most predominant *Bifidobacterium* species in the murine gastrointestinal tract, and its use in carefully designed animal studies could hold the key to identifying the host-microbiota molecular mechanisms impacting the host. Despite the availability of 386 *Bifidobacterium* whole-genome sequences in public databases (source: Genomes Online Database, queried on 1 August 2017), there has been no complete *B. pseudolongum* genome sequence obtained from a mouse isolate. The only complete *B. pseudolongum* genome sequence—for strain PV8-2 (GenBank accession number CP007457)—was isolated from feces of an anemic Kenyan infant (11).

Here, we report the complete genome sequence of *B. pseudolongum* strain UMB-MBP-01, isolated from the feces of C57BL/6J mice through passages and screening on Bifidus selective medium (BSM) agar (Sigma-Aldrich, St. Louis, MO, USA). This strain was identified in microbiome profiling studies associated with improved transplant outcome in a murine model of cardiac heterotypic transplantation E. F. Mongodin and J. S. Bromberg, unpublished data. Genomic DNA extraction was performed using a lysozyme/mutanolysin-based cell lysis followed by purification using the Wizard genomic DNA purification kit (Promega, Madison, WI, USA). Library construction (5- to 20-kb-long insert) and sequencing were performed at the University of Maryland’s Institute for Genome Sciences using one single-molecule real-time (SMRT) cell on a PacBio RS II system (Pacific Biosciences, Menlo Park, CA, USA). A total of 159,138 reads with an average length of 4,598 bp (total bases, 731,667,147 bp) were assembled into a single contig (chromosome) using the Hierarchical Genome Assembly Process (HGAP) assembler. The genome was then automatically annotated using the IGS Prokaryotic Annotation Pipeline (12). The genome of *B. pseudolongum* strain UMB-MBP-01 consists of a 2,008,102-bp circular chromosome containing 52 tRNA genes and 4 rRNA operons encoding 12 rRNA genes. The G+C content of the genome is 63.4%, and a total of 1,697 protein-coding sequences were predicted. Preliminary analyses using the BLAST score ratio comparison tool (13) showed that 271 predicted genes in the UMB-MBP-01 genome (15.96% of the genome) do not have homologs in the PV8-2 genome. This set of genes could be involved in murine host colonization and/or anti-inflammatory properties of our UMB-MBP-01 isolate.

### Accession number(s).

The *B. pseudolongum* UMB-MBP-01 complete genome sequence is available under GenBank accession number CP022544.
